# OsBIC1 Directly Interacts with OsCRYs to Regulate Leaf Sheath Length through Mediating GA-Responsive Pathway

**DOI:** 10.3390/ijms23010287

**Published:** 2021-12-28

**Authors:** Cong Li, Xin Wang, Liya Zhang, Chunyu Zhang, Chunsheng Yu, Tao Zhao, Bin Liu, Hongyu Li, Jun Liu

**Affiliations:** 1 Institute of Crop Sciences, Chinese Academy of Agricultural Sciences, Beijing 100081, China; licong202103@163.com (C.L.); WX940709@163.com (X.W.); zly13070156288@163.com (L.Z.); n_zcy@163.com (C.Z.); yuchunsheng1989@163.com (C.Y.); zhaotao02@caas.cn (T.Z.); liubin05@caas.cn (B.L.); 2 Institute of Nanfan & Seed Industry, Guangdong Academy of Sciences, Guangzhou 510316, China

**Keywords:** blue-light inhibitor of cryptochrome (BIC), cryptochrome (CRY), leaf sheath length, blue light, GA

## Abstract

Cryptochrome 1 and 2 (CRY1 and CRY2) are blue light receptors involved in the regulation of hypocotyl elongation, cotyledon expansion, and flowering time in *Arabidopsis* *thaliana*. Two cryptochrome-interacting proteins, Blue-light Inhibitor of Cryptochrome 1 and 2 (BIC1 and BIC2), have been found in *Arabidopsis*. BIC1 plays critical roles in suppressing the physiological activities of CRY2, which include the blue light-dependent dimerization, phosphorylation, photobody formation, and degradation process, but the functional characterization of BIC protein in other crops has not yet been performed. To investigate the function of BIC protein in rice (*Oryza sativa*), two homologous genes of *Arabidopsis* *BIC1* and *BIC2*, namely *OsBIC1* and *OsBIC2* (*OsBICs*), were identified. The overexpression of *OsBIC1* and *OsBIC2* led to increased leaf sheath length, whereas mutations in *OsBIC1* displayed shorter leaf sheath in a blue light intensity-dependent manner. OsBIC1 regulated blue light-induced leaf sheath elongation through direct interaction with OsCRY1a, OsCRY1b, and OsCRY2 (OsCRYs). Longitudinal sections of the second leaf sheath demonstrated that OsBIC1 and OsCRYs controlled leaf sheath length by influencing the ratio of epidermal cells with different lengths. RNA-sequencing (RNA-seq) and quantitative Real-Time Polymerase Chain Reaction (qRT-PCR) analysis further proved that OsBIC1 and OsCRYs regulated similar transcriptome changes in regulating Gibberellic Acids (GA)-responsive pathway. Taken together, these results suggested that OsBIC1 and OsCRYs worked together to regulate epidermal cell elongation and control blue light-induced leaf sheath elongation through the GA-responsive pathway.

## 1. Introduction

Cryptochromes are blue light receptors that regulate growth and development in plants [1]. In *Arabidopsis* *thaliana*, Cryptochrome 1 and 2 (CRY1 and CRY2) were first discovered to regulate hypocotyl elongation and flowering time, respectively [2,3,4]. CRY1 is the major blue light receptor that regulates hypocotyl elongation, whereas CRY2 mediates blue light-dependent hypocotyl elongation under low blue light intensity [1]. In the process of exploring cryptochrome signal transduction pathways and interaction proteins, two cryptochrome inhibitor proteins, Blue-light Inhibitor of Cryptochrome 1 and 2 (BIC1 and BIC2), have been found in *Arabidopsis* [5]. The latest research has proven that BIC1 inhibits the phenotype of CRY2 in regulating flowering time and hypocotyl elongation by suppressing the dimerization, phosphorylation, and degradation of CRY2 in a blue light-dependent manner [5,6,7]. Further investigation reveals that there is a CRY-BIC negative feedback loop to regulate blue light sensitivity in *Arabidopsis* [5,6,7]. All these results help us to elucidate the interaction mechanism between BIC and CRY in dicotyledonous plants. However, the function of BIC and the relationship between CRY and BIC in monocots plants have not been clarified.

Cryptochromes have been found throughout crop plants, including rice (*Oryza sativa*), but the regulation mechanism of CRY in crops is poorly understood [8,9,10,11,12]. Three genes in rice encode cryptochromes, referred to as *OsCRY1a*, *OsCRY1b* and *OsCRY2* [13,14,15]. Previous studies show that OsCRY1a and OsCRY1b localize in the nucleus and cytoplasm. The overexpression of *OsCRY1a*, *OsCRY1b* and *OsCRY2* result in shortened leaf sheath phenotype in rice [13]. In *OsCRY2 RNAi* plants, it was found that the down-regulation of *OsCRY2* resulted in delayed flowering time in rice [14]. To find out how OsCRYs regulate leaf sheath length and flowering time in rice, Hirose et al. obtained the *Oscry1b* mutants from the Tos17 mutant library and constructed *Oscry1a* and *Oscry2* knockdown transgenic lines using RNA interference technology [15]. Further investigation displayed that OsCRY1 activated gibberellic acids (GAs) oxidase through a blue light signal to reduce the content of active gibberellin in rice [15]. GA is a phytohormone that regulates plant seed germination, stem elongation and flowering induction [16]. As shown previously, gibberellin acids, brassinosteroids (BR), and auxin are generally implicated in promoting hypocotyl elongation and determining plant height; nevertheless, this effect is inhibited during photomorphogenic development, demonstrating that phytohormones and light are antagonistic in regulating hypocotyl development [17,18,19,20,21]. Previous studies have revealed that cryptochromes inhibited blue light-induced hypocotyl elongation through the GAs metabolic pathway [17,22,23,24]. Blue light triggers the reduction in active gibberellin content and represses two gibberellin 20-oxidase genes (*OsGA20ox2* and *OsGA20ox4*) and induces four gibberellin 2-oxidase genes (*OsGA2ox4*–*OsGA2ox7*) via *OsCRY1a* and *OsCRY1b* [24]. These results suggest that OsCRYs can regulate leaf sheath elongation through the GA response pathway, but whether other proteins are involved in this process is unclear. In the important dicotyledon crop soybean (*Glycine max*), seven cryptochrome proteins (GmCRYs, including GmCRY1a, GmCRY1b, GmCRY1c, GmCRY1d, GmCRY2a, GmCRY2b and GmCRY2c) have been identified and divided into two clades according to *Arabidopsis* CRY1 and CRY2, respectively. The studies in soybean show that GmCRYs involve in the regulation of photoperiodic flowering and leaf senescence [11,25]. Recent studies discovered that GmCRYs regulate stem elongation through crosstalk with the GA metabolic pathway [26]. All these results demonstrate that CRY can regulate plant growth through the GA-response pathway, but it is unclear whether CRY has the same function in monocotyledon plants and which proteins are involved in this process.

Here, we uncovered the function of OsBICs by analyzing the phenotype of the *Osbic1* mutant, *OsBIC1* and *OsBIC2* overexpressing lines in rice. For the first time in monocotyledonous plants, we revealed the relationship of OsBICs and OsCRYs in regulating leaf sheath length using genetic analysis and improved the regulation mechanism of OsCRYs in regulating rice growth and development via the GA-response pathway.

## 2. Results

### 2.1. OsBICs Promoted Leaf Sheath Elongation in Blue Light Specific Manner

**Figure 1:**
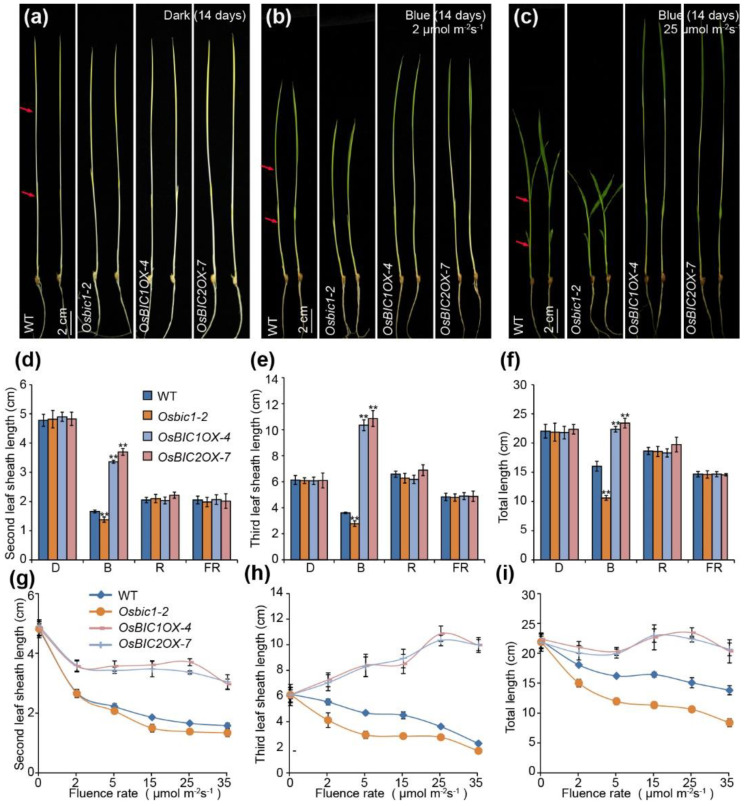
OsBICs (OsBIC1 and OsBIC2) promoted leaf sheath growth under blue light conditions. (**a**–**c**) Representative seedlings image of WT, *Osbic1-2* mutant, and *OsBICs* overexpression lines (*OsBIC1OX-4* and *OsBIC2OX-7*) grown under continuous dark (**a**), 2 µmol m^−2^s^−1^ blue light (**b**), and 25 µmol m^−2^s^−1^ blue light (**c**) conditions at 28 °C for 14 days. The red arrow represented the measured position of the second leaf sheath and the third leaf sheath. Scale bars = 2 cm. (**d**–**f**) The statistics results of second leaf sheath length (**d**), third leaf sheath length (**e**), and total length (**f**) of 14 days old seedlings grown under continuous dark, blue light (25 µmol m^−2^s^−1^), red light (25 µmol m^−2^s^−1^), or far-red light (15 µmol m^−2^s^−1^) conditions. Mean values ± s.d. (*n* = 20) are shown. Comparisons were performed by Student’s *t*-tests (** *p* < 0.01). (**g**–**i**) The statistics results of second leaf sheath length (**g**), third leaf sheath length (**h**), and total length (**i**) of 14 days old seedlings grown under continuous blue light with an intensity of 0 to 35 µmol m^−2^s^−1^. Kita-ake wild-type plants (WT) were used as control.

Using the homology alignment method, we successfully identified two genes homologous to Arabidopsis *BIC1* and *BIC2*, referred to as *OsBIC1* and *OsBIC2*. *OsBIC1* locates on chromosome 4 and encodes a protein with 217 amino acid residues. *OsBIC2* encodes a protein of 195 amino acid residues and is located on chromosome 2. To investigate the function of OsBIC1 and OsBIC2 in rice, we constructed the *Pubi:OsBIC1-3Flag* (*OsBIC1OX*) and *Pubi:OsBIC2-3Flag* (*OsBIC2OX*) overexpression vectors under the *Maize ubiquitin* 1 promoter (*Pubi*) and obtained multiple transgenic lines by *Agrobacterium*-mediated transformation. In total, 12 *OsBIC1OXs* and 9 *OsBIC2Xs* transgenic lines, which were verified by immunoblotting probed with an anti-Flag antibody, recapitulated for at least two generations demonstrating increased leaf sheath length phenotypes under blue light conditions. *OsBIC1OX-3*, *OsBIC1OX-4*, *OsBIC2OX-6*, and *OsBIC2OX-7* overexpression lines expressed the same level of exogenous OsBIC1 or OsBIC2 proteins, so two independent overexpression lines of both *OsBIC1* and *OsBIC2* (*OsBIC1OX-4* and *OsBIC2OX-7*) were chosen for further experiments (Figure S1a). The respective overexpression of *OsBIC1* and *OsBIC2* in rice under the control of the *Maize ubiquitin* 1 promoter (*Pubi*) increased leaf sheath length in response to blue light (Figure 1a–c). Two independent loss-of-function lines of *OsBIC1*, *Osbic1-1* and *Osbic1-2*, were obtained by CRISPR (clustered regularly interspaced short palindromic repeat)/Cas9-mediated genome-editing system (Figure S1b). DNA sequencing identified that both *Osbic1-1* and *Osbic1-2* contained a single base insertion in the first exon of *OsBIC1*, causing frameshifts and premature termination of protein translation (Figure S1b). Unfortunately, we did not obtain the *Osbic2* mutant, probably due to the aberrant DNA structure or base’s composition. Both *Osbic1-1* and *Osbic1-2* decreased the leaf sheath length under blue light conditions, so the *Osbic1-2* mutant was chosen for further studies. *OsBIC1OX-4*, *OsBIC2OX-7*, and *Osbic1-2* displayed opposite phenotypes in leaf sheath length under 2 µmol m^−2^s^−1^ or 25 µmol m^−2^s^−1^ blue light conditions, and the leaf sheath length of the *Osbic1* mutant was significantly shorter than that of WT, whereas the overexpression of *OsBICs* resulted in an obviously increased leaf sheath length phenotype (Figure 1a–c). To find out whether *OsBIC1* and *OsBIC2* respond to blue light specifically, WT, *OsBIC1OX-4*, *OsBIC2OX-7*, and *Osbic1-2* were grown under dark, blue, red, and far-red light conditions for 14 days, respectively. Statistical analysis showed that the second leaf sheath length, third leaf sheath length, and seedlings total length of *OsBIC1OX-4*, *OsBIC2OX-7*, and *Osbic1-2* displayed significant differences when compared with WT under blue light, but no differences were observed under continuous dark, red, and far-red light conditions, indicating that *OsBICs* promoted leaf sheath elongation in a blue light-specific manner (Figure 1d–f). To further confirm this result, WT, *OsBIC1OX-4*, *OsBIC2OX-7*, and *Osbic1-2* were grown under blue light of different intensities, ranging from 0 µmol m^−2^s^−1^ to 35 µmol m^−2^s^−1^. Similar results showed that the second and third leaf sheath lengths of *Osbic1-2* were significantly shorter than that of WT, whereas in *OsBIC1OX-4* and *OsBIC2OX-7*, the second and third leaf sheaths were obviously longer than that of WT under all blue light intensity (low to high), demonstrating that *OsBICs* participated in the regulation of leaf sheath growth regardless of low blue light or high blue light conditions (Figure 1g–i). These results suggested that *OsBIC1* and *OsBIC2* promoted leaf sheath elongation in a blue light-specific manner.

### 2.2. OsBIC1 Directly Interacted with OsCRYs to Regulate Blue Light-Induced Leaf Sheath Growth

Previous studies in rice exhibit that the overexpression of *OsCRYs* (*OsCRY1a*, *OsCRY1b,* and *OsCRY2*) inhibited the leaf sheath growth under blue light conditions [13,14,15]. Combined with the research results in *Arabidopsis*, BIC1 inhibits the physical function of CRY2 via direct interaction [5,6]. We wondered whether OsCRYs and OsBICs work together to regulate leaf sheath length in rice. *Oscry1a*, *Oscry1b,* and *Oscry2* mutants were constructed by the CRISPR/Cas9 system and identified through PCR and DNA sequencing (Figure S2a–c). Western blot showed that OsCRY1a and OsCRY2 proteins could not be detected in *Oscry1a-2* and *Oscry2-1* mutant lines, and *OsCRY1b* was partially reduced in the *Oscry1b-1* mutant. The quantitative real-time polymerase chain reaction (qRT-PCR) result displayed that the expression of *OsCRY1b* reduced compared to WT (Figure S2d,e). *Oscry1a-2* (1 bp deletion at the target 2 site), *Oscry1b-1* (4 bp deletion at the target 1 site), and *Oscry2-1* (129 bp deletion at the target 1 site) mutants caused frameshifts and premature termination of protein translation and were chosen for further experiments (Figure S2). *Oscry1a-2*, *Oscry1b-1*, and *Oscry2-1* exhibited a much longer leaf sheath than that of WT under blue-light (0–35 µmol m^−2^s^−1^), and there were no obvious differences in leaf sheath between WT and *Oscry1a-2*, *Oscry1b-1*, and *Oscry2-1* mutants under continuous dark, red, and far-red light conditions, confirming the former results that *OsCRYs* could repress the blue light-induced leaf sheath growth in rice (Figure S3). Moreover, *Oscry1a-2* and *Oscry1b-1* showed a much longer leaf sheath than *Oscry2-1* under high blue-light intensity (above 15 µmol m^−2^s^−1^), demonstrating that the contribution of *OsCRY2* in blue light-induced leaf sheath growth inhibition was limited to a low light intensity range, while *OsCRY1a* and *OsCRY1b* were considered as the major blue light receptor regulating leaf sheath growth (Figure S3). Therefore, *OsCRY1a* and *OsCRY2* were chosen for further experiments.

**Figure 2:**
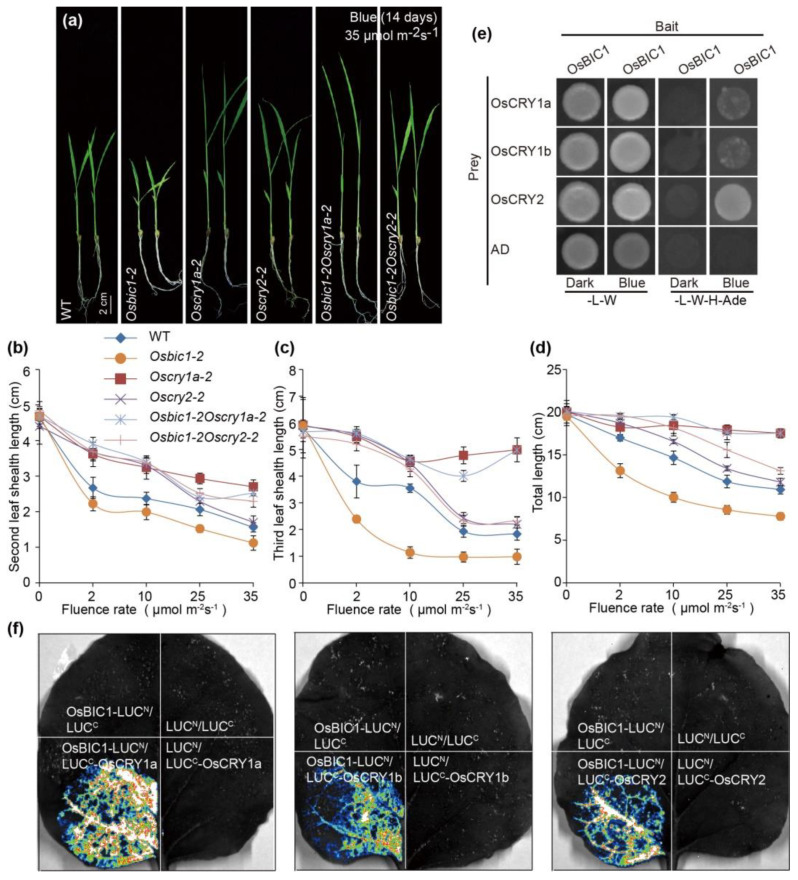
OsBIC1 directly interacted with OsCRYs (OsCRY1a, OsCRY1b, and OsCRY2) to control leaf sheath length under blue light conditions. (**a**) Representative seedlings image of WT and each indicated mutants grown under continuous 35 µmol m^−^^2^s^−^^1^ blue light conditions at 28 °C for 14 days. Scale bars = 2 cm. (**b**–**d**) The statistics results of second leaf sheath length (**b**), third leaf sheath length (**c**), and total length (**d**) of 14 days old seedlings grown under continuous blue light with intensity of 0 to 35 µmol m^−2^s^−1^. Mean values ± s.d. (*n* = 20) are shown. (**e**) OsBIC1 interacted with OsCRYs in yeast two-hybrid assays. Empty vector expressing the AD domain was negative control. (**f**) Interaction between OsBIC1 and OsCRYs under long day conditions in LCI assays.

In order to investigate the genetic relationship between *OsBIC1* and *OsCRYs*, *Osbic1Oscry1a* and *Osbic1Oscry2* double mutants were generated via crossing *Osbic1-2* with *Oscry1a-2* and *Oscry2-1*, respectively. The *Osbic1Oscry1a* and *Osbic1Oscry2* double mutants displayed long leaf sheath phenotypes compared with WT and *Osbic1-2* under 35 µmol m^−2^s^−1^ blue light conditions (Figure 2a). Similar results were observed under the increasing intensity of blue light, ranging from 0 µmol m^−2^s^−1^ to 35 µmol m^−2^s^−1^ (Figure 2b–d). *Osbic1Oscry2* had much more influence on leaf sheath length at a low blue light intensity similar to that of *Oscry2-1* (below 10 µmol m^−2^s^−1^) (Figure 2b–d). These results indicated that *OsCRY1a* and *OsCRY2* acted epistatically to *OsBIC1*, and OsCRY1a and OsCRY2 worked in the same pathway with OsBIC1 to regulate leaf sheath length. To further clarify the physical interaction between OsCRY1a, OsCRY1b, OsCRY2, and OsBIC1, we performed yeast two-hybrid assays, and the results revealed that OsBIC1 strongly interacted with OsCRY2 under blue light conditions, but the interaction between OsBIC1 with OsCRY1a and OsCRY1b appeared to be slightly weaker when compared with OsCRY2 (Figure 2e). Moreover, OsBIC2 could interact with the OsCRYs protein under both dark and blue conditions (Figure S4). To further confirm this result, we checked the interaction between OsBICs and OsCRYs in vivo by firefly luciferase complementation imaging (LCI) assays. The *pCambia-35S:OsBICs-LUC^N^* (Os*BIC1-LUC^N^*, Os*BIC2-LUC^N^*) and *pCambia-35S:OsCRYs-LUC^C^* (*OsCRY1a-LUC^C^*, *OsCRY1b-LUC^C^*, *OsCRY2-LUC^C^*) plasmids were co-transformed into *Nicotiana benthamiana* leaves, respectively. Luminescence was observed in all the combinations containing OsBIC1 and OsCRYs (Figure 2f). The LCI assays results confirmed the physical interaction between OsBICs and OsCRYs in *Nicotiana benthamiana* (Figure 2f and Figure S4). These results indicated that OsBIC1 physically interacted with OsCRYs in a blue light intensity-dependent manner, while OsBIC2 interacted with OsCRYs in a blue light intensity-independent manner (Figure 2 and Figure S4). Taken together, we concluded that OsBICs and OsCRYs influenced leaf sheath length through direct interaction. Considering the opposite phenotype of leaf sheath length between OsBICs and OsCRYs, we speculated that OsBICs inhibited the function OsCRYs in leaf sheath length regulation via interaction directly.

### 2.3. OsBIC1 and OsCRYs Regulated Leaf Sheath Length by Regulating Epidermal Cell Length

**Figure 3:**
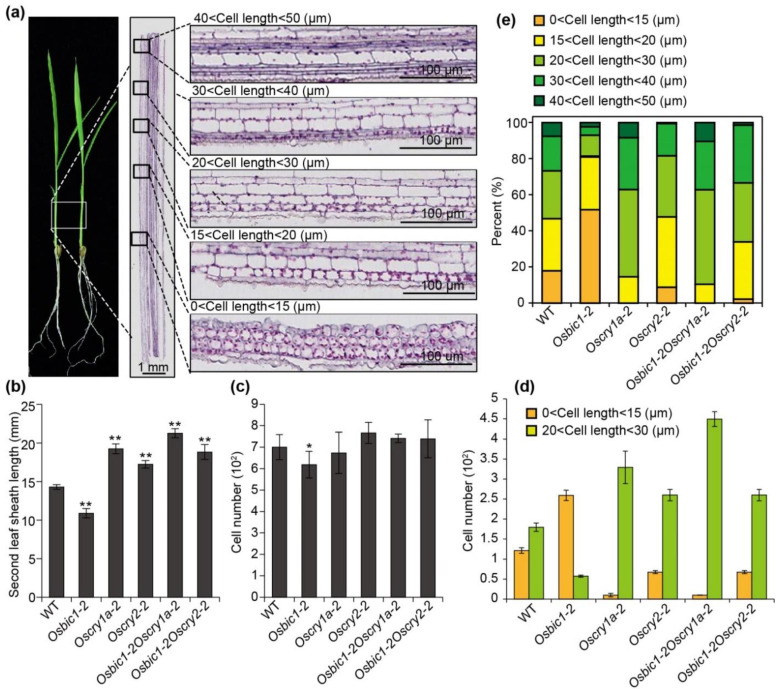
OsBIC1 and OsCRYs controlled the cell elongation in response to blue light. (**a**) Two seedlings of WT to show the position of the second leaf sheath ((**a**) left panel). The epidermal cells were divided into five groups according to the cell length: 0–15 µm, 15–20 µm, 20–30 µm, 30–40 µm, and 40–50 µm ((**a**) middle panel and right panel). (**b**–**d**) Comparisons of the second leaf sheath length (**b**), cell number of the outermost epidermal cells (**c**), and cell numbers of the outermost epidermal cells of 0–15 µm and 20–30 µm in length (**d**) analysis between WT and each indicated mutants. (**e**) Comparison of the percentage of cell number in different groups between WT and each indicated mutants. Mean values ± s.d. (*n* = 10) are shown. Comparisons were performed by Student’s *t*-tests (* *p* < 0.05, ** *p* < 0.01).

To find out the reasons that caused the differences in leaf sheath length, we performed longitudinal sections histological analysis. WT and each indicated mutant were grown under continuous blue light conditions at 28 °C for 14 days (35 µmol m^−2^s^−1^). The complete second leaf sheaths were collected and used for longitudinal sections histological analysis. Interestingly, while observing epidermal cell length, we found that the epidermal cells of the second leaf sheath gradually lengthen from bottom to top, showing a gradient in length, ranging from 10 to 50 µm (Figure 3a). Therefore, to better understand the regularity of cell length in each genotype, we divided the epidermal cells into five different groups according to the cell length: 0–15 µm, 15–20 µm, 20–30 µm, 30–40 µm, and 40–50 µm (Figure 3a, middle panel and right panel). Then, we calculated and compared the cell number in different groups and plotted the percentage of cell numbers in each group between WT and each indicated mutants (Figure 3e). The percentage results showed that *Oscry1a-2*, *Oscry2-1*, *Osbic1Oscry1a,* and *Osbic1Oscry2* showed a decrease in cell number with cell length less than 15 µm and an increase in cell number with a length more than 20 µm when compared with WT (Figure 3d,e). The number of epidermal cells less than 15 µm in *Osbic1-2* increased obviously, while cell number in cell length more than 20 µm decreased greatly in *Osbic1-2* compared with WT and other indicated mutants (Figure 3e). Subsequently, we calculated the cell number of the outermost epidermal cells in WT and each indicated mutant. Similar to our prior results, the second leaf sheath length of WT was on average longer than that of *Osbic1-2* and shorter than that of *Oscry1a-2*, *Oscry2-1*, *Osbic1Oscry1a,* and *Osbic1Oscry2* (Figure 3b). The cell number of the outermost epidermal cells showed no obvious differences in *Oscry1a-2*, *Oscry2-1*, *Osbic1Oscry1a,* and *Osbic1Oscry2* when compared with WT, and only a slight decrease in cell number was observed in *Osbic1-2* (Figure 3c). Taken together, these results suggested that longer cell length made more contribution on increasing leaf sheath length in *Oscry1a-2*, *Oscry2-1*, *Osbic1Oscry1a,* and *Osbic1Oscry2* plants, the shorter sheath length in *Osbic1-2* is attributed to a greater proportion of epidermal cells smaller than 15 µm, consolidating that *OsBIC1* and *OsCRYs* controlled leaf sheath length through influencing the ratio of epidermal cells with different lengths.

### 2.4. OsBIC1 and OsCRY1s Regulated Similar Transcriptome Changes under Blue Light Conditions

**Figure 4:**
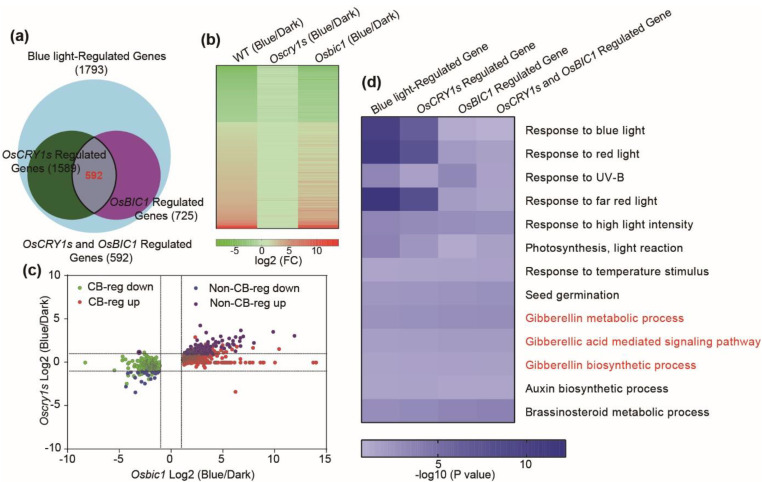
OsBIC1 and OsCRY1s regulated similar transcriptome changes under blue light conditions. (**a**) Venn diagram depicting the overlapping profiles among blue light-regulated, *OsCRY1s*-regulated, *OsBIC1*-regulated, and *OsCRY1s* and *OsBIC1*-regulated genes determined by RNA-seq. Blue light-regulated genes showed a factor of > 2 change of mRNA (Fold Change (FC) > 2, *p* value < 0.01, False Discovery Rate (FDR) < 0.01) in WT-Blue when compared with WT-Dark. The blue light-regulated genes that showed FC < 2 or *p* > 0.01 in *Oscry1s*-Blue in comparison to *Oscry1s*-Dark were defined as *OsCRY1s*-regulated genes. The blue light-regulated genes that showed FC > 2 and *p* < 0.01 in *Osbic1*-Blue in comparison to *Osbic1*-Dark were defined as *OsBIC1*-regulated genes. The overlapping profiles among blue light-regulated, *OsCRY1s*-regulated, and *OsBIC1*-regulated genes were defined as CB-reg genes. (**b**) Hierarchical clustering analysis of the CB-reg genes in blue light-regulated, *OsCRY1s*-regulated, and *OsBIC1*-regulated genes profiles. Green indicated down-regulation genes, and red was used to indicate up-regulation genes. (**c**) Scatter plots show the correlation between the *Osbic1* blue/dark log2 (FC) and *Oscry1s* blue/dark log2 (FC). The dashed lines indicated log2 (FC) = 1 and −1. (**d**) Enriched gene ontology functional categories in the blue light-regulated, *OsCRY1s*-regulated, *OsBIC1*-regulated, and *OsCRY1s* and *OsBIC1*-regulated genes.

To investigate the mechanism of *OsBIC1* and *OsCRYs* in regulating cell elongation, the *Oscry1aOscry1b* (*Oscry1s*) double mutant was generated via crossing *Oscry1a-2* and *Oscry1b-1*, and the etiolated seedlings of WT, *Osbic1*, *Oscry1s* were kept in the dark (dark treatment, WT-Dark, *Osbic1*-Dark, and *Oscry1s*-Dark) or exposed to blue light 2 h (blue light treatment, WT-Blue, *Osbic1*-Blue, and *Oscry1s*-Blue) to conduct high-throughput RNA-sequencing assays. Compared with WT-dark, 1793 differentially expressed genes (Fold Change (FC) > 2, *p* value < 0.01, and False Discovery Rate (FDR) < 0.01) were identified in the WT-Blue plants and defined as blue light-regulated genes, in which 1286 and 507 genes were up- and down-regulated, respectively (Figure 4a). Out of the 1793 blue light-regulated genes, 1589 were not differentially expressed in *Oscry1s*-Blue plants when compared with *Oscry1s*-Dark and defined as *OsCRY1a* and *OsCRY1b* (*OsCRY1s*)-regulated genes (FC < 2 or *p* > 0.01) (Figure 4a). Out of 1793 blue light-regulated genes, 725 were differentially expressed in the *Osbic1*-blue plants when compared with *Osbic1*-dark, and named as *OsBIC1*-regulated genes (FC > 2, *p* value < 0.01, and FDR < 0.01) (Figure 4a). Venn diagram analysis showed that 592 candidate genes were detected in the overlapping profiles among blue light-regulated, *OsCRY1s*-regulated, and *OsBIC1*-regulated genes, defined as *OsCRY1s* and *OsBIC1*-regulated (CB-reg) genes (Figure 4a). Then we performed hierarchical clustering analysis of the CB-reg genes and found that 386 *OsCRY1s* and *OsBIC1*-regulated genes were up-regulated, and 206 genes were down-regulated in WT and *Osbic1-2* in response to blue light (Figure 4b). Then, we analyzed the correlation between OsCRY1s and OsBIC1 regulated genes by using scatter plots analysis. The red dots represented up-regulation CB-reg genes, and green dots indicated down-regulation CB-reg genes. The purple and blue dots represented up-regulation or down-regulation genes that were not co-regulated by OsCRY1s and OsBIC1 (Non-CB-reg) (Figure 4c). Taken together, nearly 82% (592 out of 725) of the *OsBIC1*-regulated genes were also regulated by *OsCRY1s*, demonstrating that *OsBIC1* and *OsCRY1s* regulated similar transcriptome changes under blue light conditions and *OsBIC1* controlled blue light-induced leaf sheath growth dependent on *OsCRY1s* (Figure 4a–c). To further investigate the genetic regulatory pathway associated with the leaf sheath growth in the *Osbic1-2* and *Oscry1s* plants, gene ontology (GO) was used to analyze the blue light-regulated, *OsCRY1s*-regulated, *OsBIC1*-regulated, and *OsCRY1s* and *OsBIC1*-regulated genes. GO analysis revealed that genes regulated by *OsBIC1* and *OsCRY1s* in response to blue light were mainly enriched in the pathways related to response to blue light, red light, high light intensity, seed germination and so on, indicating that *OsBIC1* and *OsCRY1s* were closely related to light response (Figure 4d). It was important to note that the genes were also enriched in the gibberellin metabolic process, gibberellic acid-mediated signaling pathway, gibberellin biosynthetic process, auxin biosynthetic process, and brassinosteroid metabolic process (Figure 4d). GA increases rice internode length by promoting cell division and cell elongation in rice, suggesting that *OsBIC1* and *OsCRY1s* are involved in the regulation pathway of the hormone in rice, especially GA (Figure 4d).

### 2.5. OsBIC1 and OsCRYs Regulated Leaf Sheath Length through the GA-Responsive Pathway

**Figure 5:**
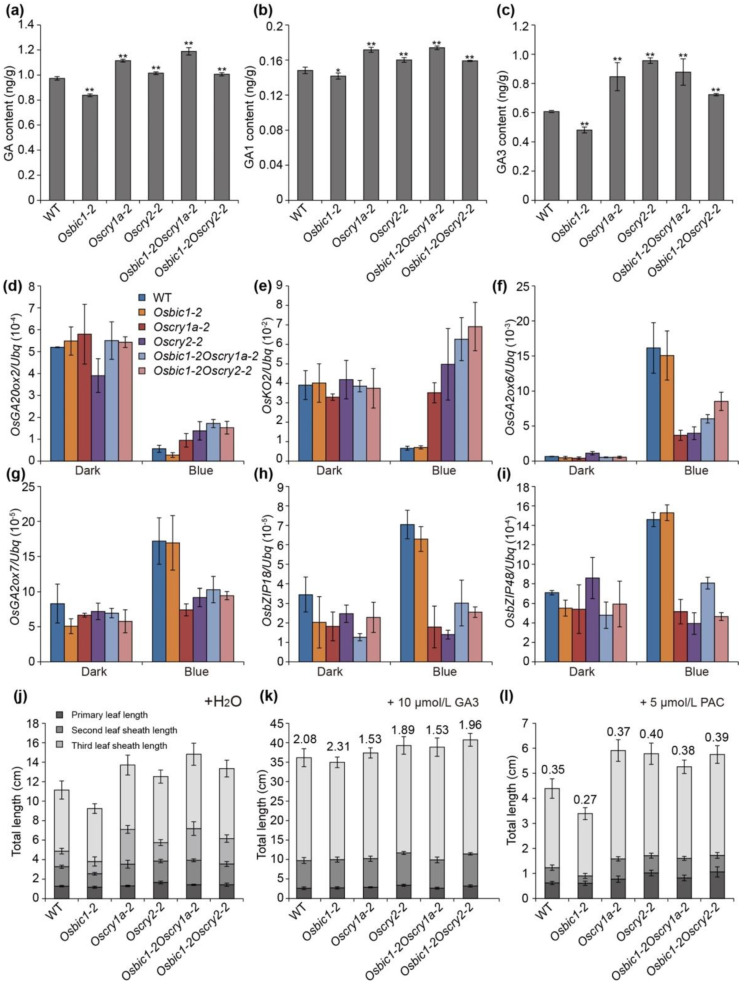
*OsBIC1* and *OsCRYs* regulated the gibberellic acid (GA)-responsive gene expression under blue light conditions. (**a**–**c**) The GA, GA1, and GA3 content of seven-day-old seedlings grown in continuous blue light conditions (25 µmol m^−2^s^−1^). Comparisons were performed by Student’s *t*-tests (* *p* < 0.05, ** *p* < 0.01). (**d**–**i**) Comparisons of transcription levels of GA-responsive gene using qRT-PCR between WT and each indicated mutants. The relative expression levels (REL) are shown as means ± s.d. (*n* = 3). *Ubiquitin* (*Ubq*) was used as an internal control, and the REL of the WT sample in the dark was arbitrarily set to 1. (**j**–**l**) Comparisons of leaf sheath length between WT and each indicated mutants in response to GA3 or Paclobutrazol (PAC). The leaf sheath length analyses of 14 days old seedlings grown under continuous 35 µmol m^−2^s^−1^ blue light. The number on the column indicated sensitivity to GA3 or PAC. Each number on the plot represented leaf sheath elongation ratio, which was equal to leaf sheath length under GA3 or PAC treatments divided by leaf sheath length treated with water.

In our RNA sequencing results, a series of genes related to gibberellin metabolism, gibberellin biosynthesis, and gibberellin signal transduction were differentially expressed when the etiolated seedlings of WT were exposed to blue light (Figure S5). Consistent with previous studies, quantitative reverse transcription-polymerase chain reactions (qRT-PCR) results showed that most gibberellin biosynthetic genes (*OsGA20ox2*, *OsGA3ox2*, and *OsKO2*) were down-regulated in response to blue light, while gibberellin metabolic gene (*OsGA2ox6* and *OsGA2ox7*) and gibberellic acid-mediated signaling genes (*OsbZIP18* and *OsbZIP48*) were both up-regulated (Figures S5 and S6) [24]. To confirm whether *OsBIC1* and *OsCRY1s* could participate in the GA regulation pathway, we firstly examined the endogenous bioactive levels of GAs (total GA content, GA1 content and GA3 content) in WT and indicated mutants. The total GA, GA1, and GA3 content significantly increased in *Oscry1a-2*, *Oscry2-1*, *Osbic1Oscry1a,* and *Osbic1Oscry2*, whereas drastically reduced in *Osbic1-2* compared with WT under blue light conditions, suggesting that *OsBIC1* and *OsCRY1s* involved in the regulation of GA pathway (Figure 5a–c).

To elucidate the molecular mechanism of *OsBIC1* and *OsCRYs* in controlling GA-responsive pathway, we conducted qRT-PCR to test the GA-responsive gene in WT and each indicated mutant. The transcriptional expression of the GA-responsive genes displayed no significant differences between WT and each indicated mutant under dark conditions, whereas significant change was discovered under blue light conditions (Figure 5d–i). The *OsGA20ox2* (*SD1*) and *OsKO2* (*D35*) loss of function mutants exhibit remarkable dwarf or semi-dwarf phenotypes caused by a defective early step of gibberellin biosynthesis [27,28,29,30]. The expression of *OsGA20ox2* and *OsKO2* were up-regulated in *Oscry1a-2* and *Oscry2-1* compared with WT, whereas down-regulated in *Osbic1-2* (Figure 5d,e). Contrary to the GA biosynthetic genes, the transcription levels of GA catabolic genes *OsGA2ox6* and *OsGA2ox7* in *Oscry1a-2* and *Oscry2-1* significantly reduced (Figure 5f,g) [16,31,32]. GA-mediated signaling genes play an important role in the regulation of plant height growth [20,33,34]. *OsbZIP18* and *OsbZIP48* are the *AtHY5* transcription factor homologs in rice, and the overexpression of *OsbZIP48* leads to semi-dwarf phenotypes by being involved in the regulation of GA biosynthesis and signaling [35]. The expression of *OsbZIP18* and *OsbZIP48* were down-regulated in *Oscry1a-2* and *Oscry2-1*, whereas it was up-regulated in *Osbic1-2* mutant (Figure 5h,i).

In addition, we also found that *Oscry1a-2* and *Oscry2-1* exhibited a slightly lower leaf sheath elongation ratio compared with WT, after exogenous 10 μmol/L GA3 treatment (Figure 5k). However, *Osbic1-2* showed significantly more sensitive responsiveness to GA3 than WT. Meanwhile, after exogenous 5 µmol/L Paclobutrazol (PAC) treatments, the leaf sheath elongation ratio in WT was slightly higher than that of *Osbic1-2* and lower than that of *Oscry1a-2* and *Oscry2-1* (Figure 5l). *Osbic1Oscry1a* and *Osbic1Oscry2* displayed similar results compared with *Oscry1a-2* and *Oscry2-1,* respectively (Figure 5j–l). It was worth noting that regardless of GA3 or PAC treatment, *Oscrys* and *Osbic1* always exhibited a difference in the phases of leaf sheath growth compared with WT, suggesting that *OsBIC1* and *OsCRYs* were essential for GA response and could participate in the regulation of GA signaling. These results indicated that *OsBIC1* and *OsCRYs* worked together in the same GA-responsive pathway and *OsCRYs* acted epistatically to *OsBIC1*.

## 3. Discussion

BICs are newly identified nuclear proteins and widely found in moss, gymnosperm, and angiosperm that can directly interact with CRYs to inhibit their function by blocking blue light-dependent cryptochrome dimerization [5]. *BICs* are induced by blue light, and the overexpression transgenic lines exhibit similar phenotypes resembling that of the *cry1cry2* mutant, including blue light-insensitive hypocotyl growth and delayed floral initiation in long-day (LD) photoperiod [5,6,36]. The function of *BICs* in rice or other crops is largely unknown. Here, we reported that there were two OsBICs nuclear proteins in rice, which contained a conserved CRY-interacting domain (CID) in the C-terminal region, and the transcriptional expression of *OsBICs* were induced by blue, red, and far-red light (Figures S5 and S6). *OsBICs* overexpression transgenic lines exhibited significantly blue light promotion of leaf sheath growth phenotypes, while the leaf sheath phenotype of the *Osbic1* mutant was contrary to the overexpression of *OsBICs* (Figure 1). Further studies showed that OsBICs inhibited the function of OsCRYs in regulating leaf sheath growth by interacting with OsCRYs proteins (Figure 2 and Figure 3). To summarize, there were many similarities between *OsBICs* and *Arabidopsis BICs* in terms of protein conservative domains, blue light-induced expression, subcellular localization, and the regulation of blue light-induced leaf sheath growth, suggesting that *BICs* are widely distributed in the plant, exhibited a conservative trend during biological evolution and might be involved in a similar function in other crops.

Plant height is an important character determined plant architecture, yield and stress response in rice and is controlled by phytohormones and their integrated signaling networks [37]. Gibberellins (GAs) increase plant height through mediating cell division and cell elongation in rice, and GA-deficient or GA-insensitive mutants display a dwarf or semi-dwarf phenotype [30]. Several GA biosynthesis, metabolic, and signaling-related genes have been identified to involve in the regulation of plant height, organ development, yield and stress response in rice, including *OsGA20ox2*, *OsGA3ox2*, *OsKO2*, *OsGA2ox6*, *SLR1*, and *GID1* [27,28,29,38,39]. The latest research suggests that cryptochrome and phytochrome cooperatively but independently rapidly decrease gibberellin activity in rice following light irradiation [17,22,24]. Consistent with previous results, here, we discovered that *OsBIC1* and *OsCRYs* regulated cell elongation by changing the content of active gibberellin through mediating the transcript level of gibberellin biosynthesis and metabolic genes, and the expressions of *OsGA20ox2*, *OsKO2*, *OsGA2ox6,* and *OsGA2ox7* were changed in each indicated mutant under blue light conditions (Figure 5, Figures S5 and S6). Gibberellins are primarily synthesized in actively growing and elongation tissues, *OsGA20ox2*, *OsGA3ox2*, and *SLR1* are highly expressed in inflorescence meristems [40]. Previous results show that Cytokinins (CKs) and GAs are antagonistic to regulate meristem activity [41]. The dynamic balance among auxin, GAs, and CKs determines meristem activity in inflorescence [42]. When *OsBIC1OX* and *OsBIC2OX* overexpression lines transited from the vegetative growth stage to the reproductive growth stage, both transgene lines showed taller, later-flowering, and wider leaf phenotypes compared with wild type (unpublished data). We speculated that these phenotypes might be related to the regulation of meristem activity by GA. The expression of *OsGA20ox2* and *OsKO2* increased under blue light in *Oscry1a-2*, *Oscry2-1, Osbic1Oscry1a,* and *Osbic1Oscry2* mutants compared with WT (Figure 5d,e), which might enhance the meristem activity and lead to the phenotype changing, such as leaf color and thickness. The overexpression of *OsBIC1OX* and *OsBIC2OX* inhibited the function of OsCRY1 and produced a phenotype similar to *Oscry1a-2*, *Oscry2-1, Osbic1Oscry1a,* and *Osbic1Oscry2,* which displayed an opposite phenotype to *Osbic1* (Figure 2 and Figure S3). These results will provide new ideas for us to study the regulation of meristem activity by blue light.

OsbZIP48, an HY5 Transcription Factor orthologue, directly binds to the promoter of *OsKO2* through a G-Box element and involves in GA biosynthesis in rice. The overexpression of *OsbZIP48* can significantly inhibit the expression of *OsGA20ox2* and *OsKO2* [35]. In our study, we found that the transcript level of *OsbZIP48* was down-regulated in *Oscry1a-2* and *Oscry2-1* mutants, whereas up-regulated in *Osbic1-2* mutant under blue light conditions, suggesting that *OsBIC1* and *OsCRYs* may regulate GA biosynthesis through the *OsbZIP48* gene in blue light (Figure 5). In the meanwhile, combining the previous conclusions, which was that the HY5 of *P. sativum* binds to the promoter of GA catabolism gene *GA2ox2* to promote its expression, in *Arabidopsis*, AtHY5 binds to the promoter of *AtGA2ox2* and participates in the regulation of the gibberellin metabolism [43,44]. Two CACGTG motifs in the *OsGA2ox6* promoter region (−104 and −2336) and four CACGTG motifs in the *OsGA2ox7* (+624, +1326, +1937 and +2863) genomic region were identified, speculating that *OsbZIP48* might directly bind to *OsGA2ox6* and *OsGA2ox7* genes to increase their expression. *OsBIC1* and *OsCRYs* may also control GA metabolic program through the *OsbZIP48* gene in blue light, which needs further study.

*OsbZIP48* involves in the GA signaling pathway by down-regulated the expression of *OsSLR1*, *OsSLRL1*, *OsGID1,* and *OsGID2*, which are central repressors or positive regulators of GA signaling [35,45]. The rice slender mutant (*slr1-1*) results in a constitutive gibberellin response phenotype and has very rapid extension growth in the seedling and is sterile [46]. GID1 protein acts as a positive regulator of gibberellin signaling, and the GA-insensitive dwarf mutant of rice (*gid1*) cannot respond to gibberellin [20]. Moreover, GID1 protein can specifically bind radiolabeled gibberellin and that GID1 binding to SLR1 is gibberellin-dependent [20]. However, *OsBIC1* and *OsCRYs* could not influence the expression of *OsSLR1*, *OsSLRL1*, *OsGID1,* and *OSGID2* in our experiment (Table S2), demonstrating that GA signaling genes (*OsSLR1*, *OsSLRL1*, *OsGID1* and *OSGID2*) were not controlled by blue light at the transcriptional level and *OsbZIP48* had no obvious effect on the GA signaling genes under blue light conditions (Table S2). Recent studies show that DELLA proteins, central repressors in GA signaling, are ubiquitinated by COP1 in vitro through physically interacting with COP1, which are enhanced by the COP1–SPA1 complex, suggesting that DELLA proteins are destabilized not only by the canonical GA-dependent pathway but also by COP1 [47]. In the CRYs signal transduction pathway, CRY1 physically interacts with SPA1 in a blue-light-dependent manner, and the CRY1–SPA1 interaction negatively regulates COP1 by promoting the dissociation of COP1 from SPA1 in *Arabidopsis* [48,49,50,51]. CRY2 also interacts with SPA1 under blue light conditions to suppress COP1-dependent proteolysis through stimulating the CRY2–COP1 interaction in *Arabidopsis* [52,53,54]. In rice, OsCRY1b can interact with OsCOP1 to regulate leaf sheath and flowering time in rice through the OsCOP1 pathway [15]. Our GA3 or PAC treatment experiments indicated that *OsBIC1* and *OsCRYs* were essential for GA response and could participate in the regulation of GA signaling (Figure 5). Based on the above results, we speculate that *OsBIC1* and *OsCRYs* were considered to participate in the GA signaling pathway at the protein level through the COP1–SPA1 complex. All these speculations need to be further proofed.

## 4. Materials and Methods

### 4.1. Primers and Accession Numbers

All primers used in this study were listed in Table S1. Sequence data were downloaded from the MSU Rice Genome Annotation Project database (http://rice.plantbiology.msu.edu, accessed on 30 October 2021) [55]. The accession numbers are *OsBIC1* (*LOC_Os04g33610*), *OsBIC2* (*LOC_Os02g32990*), *OsCRY1a* (*LOC_Os02g36380*), *OsCRY1b* (*LOC_Os04g37920*), *OsCRY2* (*LOC_Os02g41550*), *OsGA20ox2* (*LOC_Os01g66100*), *OsKO2* (*LOC_Os06g37364*), *OsGA3ox2* (*LOC_Os01g08220*), *OsGA2ox6* (*LOC_Os04g44150*), *OsGA2ox7* (*LOC_Os01g11150*), *OsbZIP18* (*LOC_Os02g10860*), *OsbZIP48* (*LOC_Os06g39960*), *GPA1* (*LOC_Os05g26890*), *OsKAO* (*LOC_Os06g02019*), and *ubq* (*LOC_Os03g13170*). The RNA-seq data were deposited in the National Genomics Data Center with GSA accession number CRA005544 (https://ngdc.cncb.ac.cn/?lang=en, accessed on 30 October 2021).

### 4.2. Plasmid Construction, Plant Materials and Growth Conditions

To generate *OsBIC1* and *OsBIC2* overexpression lines, the Coding DNA sequence (CDS) of *OsBIC1* (651 bp) and *OsBIC2* (585 bp) were amplified from the cDNA of young seedlings of Nipponbare wild type and inserted into the pHCF vector at the *Pst*I site using the Infusion^®^ HD Cloning Kit (Clontech, 639650), respectively [56]. To obtain the CRISPR/Cas9-engineered mutants, gRNA recognition sites of different genes were designed using the CRISPR-P website (http://crispr.hzau.edu.cn, accessed on 30 October 2021) and inserted into the pCas9-OsU3-sgRNA vector at the *Xbal*I site using the Infusion^®^ HD Cloning Kit. The above expression plasmids were individually introduced into the *Agrobacterium tumefaciens* strain EHA105 via electroporation and then transformed into Kita-ake (*Oryza sativa* L. subsp. *Japonica*) wild-type plants [57]. Wild-type (Kita-ake) and indicated transgenic rice were cultivated in a field in Beijing (39°54′ N, 116°23′ E) under natural conditions from May to October (sunny day, light intensity > 500 µmol m^−2^s^−1^). To confirm the leaf sheath length phenotypes under different light conditions, wild-type and indicated transgenic rice were grown under continuous dark, blue light, red light, or far-red light conditions in a controlled growth chamber (28 °C) for 14 days. The anti-OsCRY1a, anti-OsCRY1b, and anti-OsCRY2 polyclonal antibodies were generated by inoculating rabbits with TF-His-OsCRY1a (524–655 aa), TF-His-OsCRY1b (528–650 aa), and TF-His-OsCRY2 (407–651 aa) recombination protein, respectively. The protein of OsCRY1a (524–655 aa), OsCRY1b (528–650 aa), and OsCRY2 (407–651 aa) were expressed by using *pCold*-*TF* vector from TaKaRa (Cat#3365), the antibody was made in ABclonal Biotechnology Ltd. (Wuhan, China).

### 4.3. Yeast Two-Hybrid Assays

The yeast two-hybrid assay was performed according to the manufacturer’s instructions (ProQues two-hybrid system with Gateway technology, Invitrogen, Waltham, MA, USA). The coding DNA sequences (CDSs) of *OsBIC1* and *OsBIC2* were individually inserted into the bait vector pBridge that had been digested with *EcoR*I and *BamH*I using the Infusion^®^ HD Cloning Kit. The CDSs of *OsCRY1a*, *OsCRY1b,* and *OsCRY2* were individually fused in the prey vector pGADT7 at the *EcoR*I and *BamH*I sites using the Infusion^®^ HD Cloning Kit. The bait and prey plasmids were co-transformed into the yeast strain AH109 to test their interactions. Cultures containing the fusion protein were plated onto SD/-Tryptophan/-Leucine (SD/-L-W) and SD/-Leucine/-Tryptophan/-Histidine/-Adenine (SD/-L-W-H-Ade) plates and allowed to grow for 48 h before being photographed. The empty vector (BD) was used as a negative control.

### 4.4. Firefly Luciferase Complementation Imaging Assays in N. benthamiana

The CDS of *OsBIC1* and *OsBIC2* were individually inserted into the pCambia1300-LUC^N^ vector at the *Kpn*I and *Sal*I sites. The CDS of *OsCRY1a*, *OsCRY1b* and *OsCRY2* were individually cloned into the pCambia1300-LUC^C^ vector at the *Bgl*II and *Mlu*I sites. The LUC^N^ and LUC^C^ plasmids were individually introduced into *Agrobacterium tumefaciens* strain EHA105 via electroporation and then infiltrated into *N. benthamiana* leaves with various combinations. The assays were performed according to the previous description [58]. A low-light cooled charge-coupled device camera (Tanon 5200, Beijing, China) was used to capture the LUC image. The exposure time of LUC was 10 min for the images.

### 4.5. Longitudinal Sections Histological Analysis

WT and each indicated mutant were grown under continuous blue light conditions at 28 °C for 14 days (35 µmol m^−2^s^−1^). The second leaf sheath was fixed in FAA solution (60% (*v/v*) ethanol, dehydrated through a series of graded ethanol concentrations, and, finally, embedded in paraffin. Tissue sections were cut with a Leica rotary microtome, stained with Hematoxylin solution, and then imaged under a light microscope.

### 4.6. Sample Collection and qRT-PCR Analysis

Seeds of wild-type (WT) and indicated transgenic rice were germinated for 2 days on wet filter paper in Petri-dishes at 37 °C. The uniformly germinated seeds were picked up and sown into bottomless 96-well plates and hydroponically grown (distiller water with 1/10 MS). To complete the transcription analysis of GA-responsive genes, etiolated seedlings were grown under continuous dark conditions for seven-day and then exposed to blue light (25 µmol m^−2^s^−1^) or kept in the dark for 2 h to be collected. Samples were named as blue light treatment plants and dark treatment plants, respectively. Three independent biological replicates were analyzed, and three replicate reactions were used for each sample. All the above samples were used to extract total RNA using TRIzol reagent (Invitrogen). The first complementary DNA and cDNA were synthesized from DNase-treated total RNA (3 μg, reaction total volume 20 μL) using a TransScript^®^II One-Step gDNA Removal and cDNA Synthesis SuperMix kit (TransGen Biotech, AT311, Beijing, China). qRT-PCR was performed in 96-well optical plates using an SYBR Green RT-PCR kit (Takara, RR420A) and a Roche Light Cycler 480. *Ubiquitin* (*Ubq)* was not the blue light-regulated gene (fold change < 2 or *p* value > 0.01). Therefore, the value of gene expression was normalized to *Ubq* (Table S3).

### 4.7. RNA-seq and Data Analysis

To complete the high-throughput RNA-sequencing assay, etiolated seedlings were grown under continuous dark conditions for seven days and then exposed to blue light (25 µmol m^−2^s^−1^) or kept in the dark for 2 h to be collected for RNA extraction. The sequencing library was constructed following the manufacturer’s instructions (Illumina Inc., San Diego, CA, USA). Paired-end sequencing libraries with an insert size of approximately 200 bp were sequenced on an Illumina HiSeq 2000 sequencer at the BIOMARKER Company in Beijing. RNA-seq clean reads of three biological replicates were mapped to the *O. sativa* ssp. *japonica* reference genome after removing adaptor and low-quality nucleotides byTopHat [59]. The expression value was calculated in FPKM (fragments per kilobase of exon model per million mapped fragments), and differentially expressed genes were defined as those that showed a factor of >2 change of mRNA (fold change (FC) > 2, *p* value < 0.01, False discovery rate (FDR) < 0.01). Three independent biological replicates were analyzed, and three replicate reactions were used for each sample. To validate the utility and accuracy of RNA-seq, 18 out of 1793 blue light-regulated genes were chosen to test through qRT-PCR, which accounted for a 1% fraction of the total differentially expressed genes. The FPKM values of 18 validations genes ranged from 1 to 200 (low expression to high expression), and the fold change ranged from −250 to 250 (down-regulated and up-regulated) in WT-Dark RNA-seq data (Figures S5 and S6). Four out of the eighteen validations genes were related to blue light response (*OsBIC1*, *OsBIC2*, *OsSPA1*, and *OsCOP1*). Seven out of the eighteen validations genes were related to GA-responsive (*OsGA20ox2*, *OsKO2*, *OsGA3ox2*, *OsGA2ox6*, *OsGA2ox7*, *OsbZIP18*, and *OsbZIP48*). Seven of the eighteen validations genes remained unknown (*LOC_Os01g47370*, *LOC_Os02g04510*, *LOC_Os03g12700*, *LOC_Os06g07914*, *LOC_Os03g61160*, *LOC_Os02g37330*, and *LOC_Os04g55210*). The fold change trend of 18 genes in qRT-PCR was consistent with that in RNA-seq data (Figures S5 and S6). Moreover, Correlation analysis of fold change further confirmed that the relationship was linear between RNA-seq and qRT-PCR data, of which the correlation value (R2) was 0.8316 (Figure S7).

### 4.8. GA Content Analysis Assays

The GA content assay was performed following the manufacturer’s instructions of plant GA determination detection ELISA Kit. WT and each indicated mutants were grown under continuous blue light conditions at 28 °C for 14 days (35 µmol m^−2^s^−1^). One gram of fresh leaves was harvested and ground into powder in liquid nitrogen. Then, tissues were resuspended in 9 mL PBS buffer (pH 7.2–7.4). A sample resuspension of 10 μL and 40 μL sample dilution were added to wells together, incubated for 30 min at 37 °C, repeatedly rinsed with wash solution. A total of 50 μL HRP-Conjugate reagent was added to each well, incubated for 30 min at 37 °C, repeatedly rinsed with wash solution. Then, 50 μL chromogen solution A and chromogen solution B were added to each well, evading the light preservation for 10 min at 37 °C. Finally, 50 μL stop solution was added to each well to stop the reaction (the blue color changes to yellow color). An enzyme standard instrument under 450 nm wavelength determined absorbance (OD value) and calculated the sample concentration.

## 5. Conclusions

Our work identified the genetic contribution of OsBICs to both leaf sheath elongation and plant height growth. Our results demonstrated that *OsBIC1* and *OsCRYs* worked together to regulate epidermal cell elongation and control blue light-induced leaf sheath elongation through the GA-responsive pathway.

## Data Availability

The data presented in this study are available on request from the corresponding author.

## Supplementary Materials

The following are available online at https://www.mdpi.com/article/10.3390/ijms23010287/s1.
