# Supplementary material for: OsBIC1 Directly Interacts with OsCRYs to Regulate Leaf Sheath Length through Mediating GA-Responsive Pathway
Source: Int J Mol Sci. 2021 Dec 28;23(1):287. doi: 10.3390/ijms23010287 (PMC8745657; doi:10.3390/ijms23010287)
Supplement: Supplementary file 1 [file ijms-23-00287-s001.zip › ijms-1466191-supplementary/Supplementary Figure.pdf]

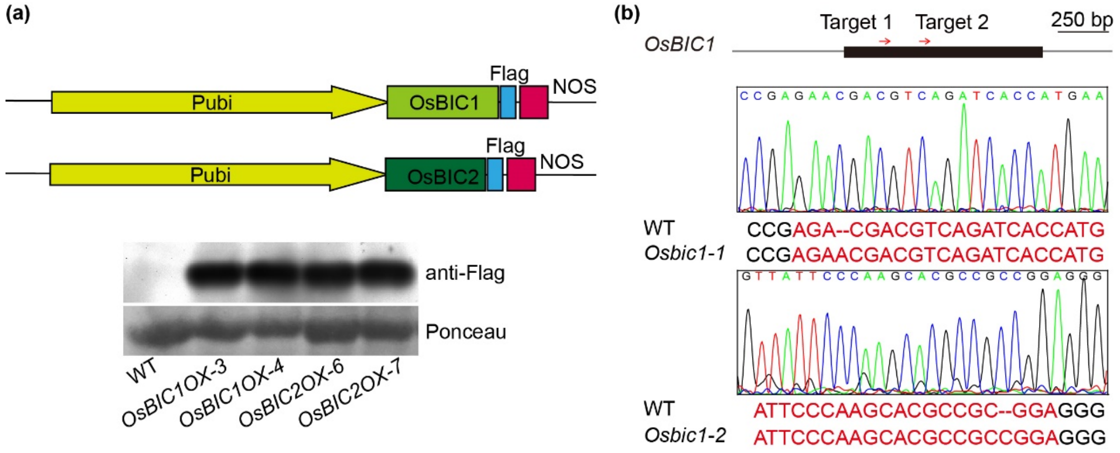

Figure S1. The representative sequences diagram and immunoblot analysis between WT and each indicated plants. (a) Protein expression analysis of OsBIC1 and OsBIC2 in transgenic plants. The immunoblot was probed with anti-Flag antibody. (b) The representative sequences of two homozygous mutants (*Osbic1-1* and *Osbic1-2*) were identified from two single-guide RNAs targets transgenic plants. The target sites of gRNA are highlighted in red letters. The black letters and dashed lines within the target sites denote nucleotide insertion and deletion respectively**.**

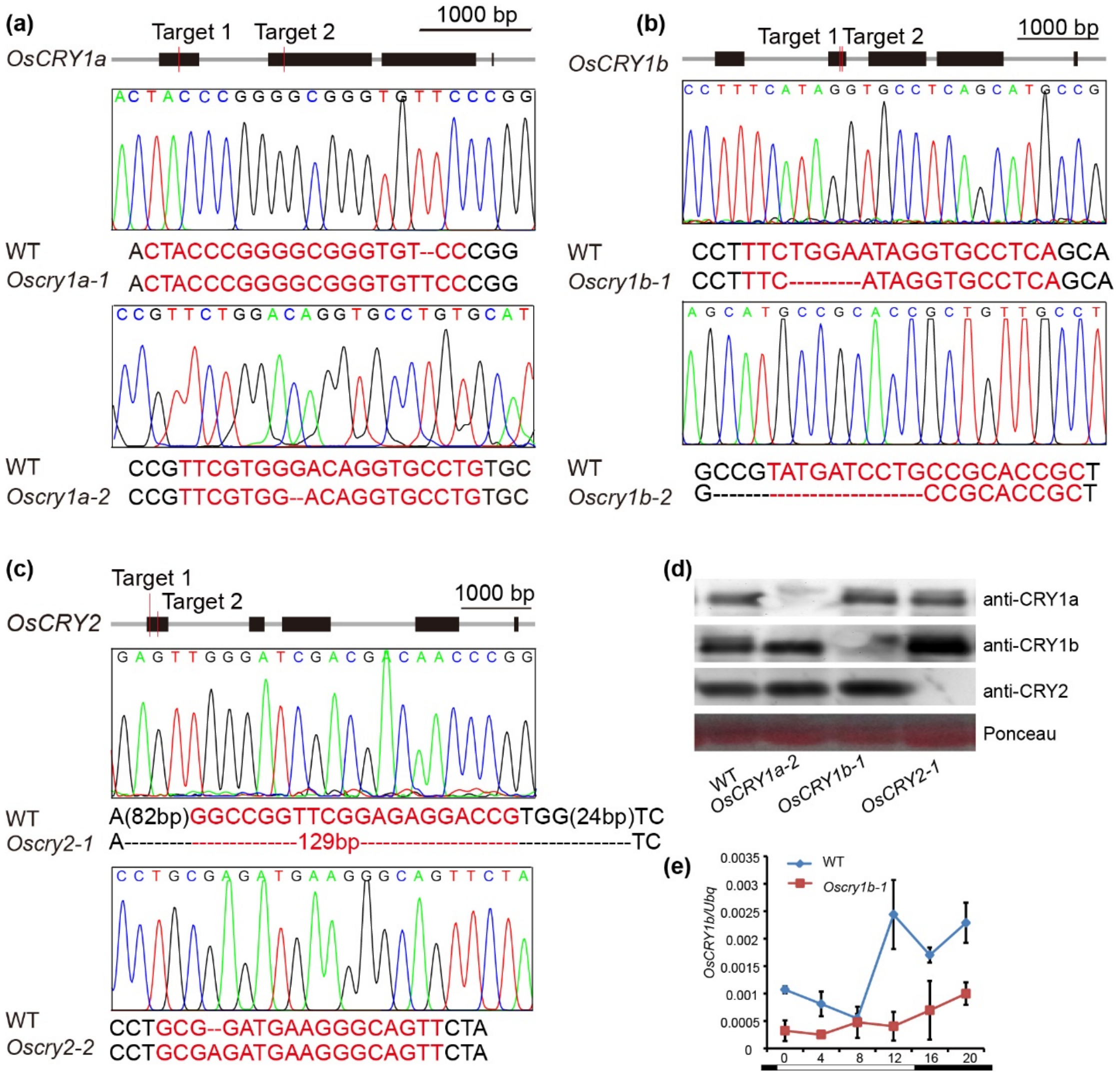

Figure S2. The representative sequences between WT and each indicated mutant plants. (a-c) The representative sequences of two homozygous mutants (*Oscry1a-1* and *Oscry1a-2*, *Oscry1b-1* and *Oscry1b-2*, *Oscry2-1* and *Oscry2-2*) were identified from two single-guide RNAs targets transgenic plants. The target sites of gRNA are highlighted in red letters. The black letters and dashed lines within the target sites denote nucleotide insertion and deletion, respectively**.** (d) Protein expression analysis of OsCRYs in *Oscry1a-2*, *Oscry1b-1* and *Oscry2-2* mutants. (e) qRT-PCR analysis of *OsCRY1b* expression in *Oscry1b-1* mutant.

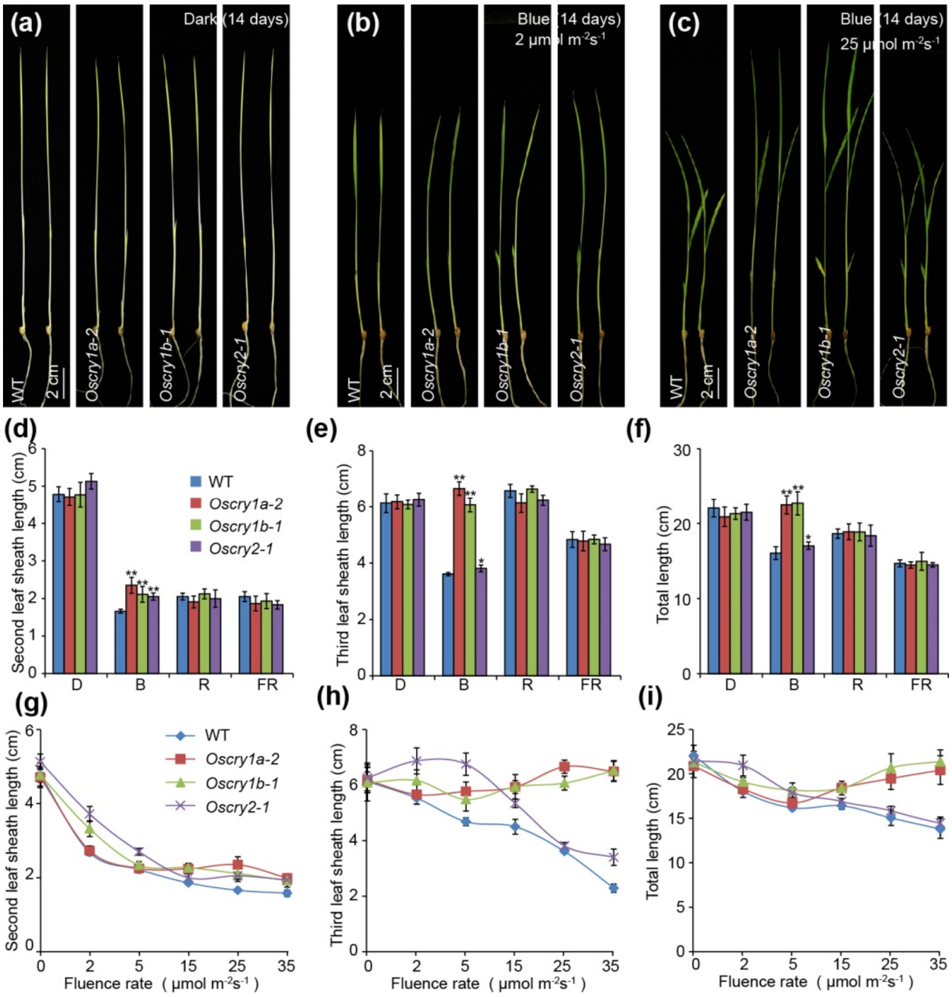

Figure S3. *OsCRYs* inhibited leaf sheath growth under blue light conditions. (a-c) Representative seedlings image of WT, *Oscry1a-2*, *Oscry1b-1* and *Oscry2-2* mutants grown under continuous dark (a), 2 μmol $m^{-2}s^{-1}$ blue light (b) or 25 μmol $m^{-2}s^{-1}$ blue light (c) conditions at 28°C for 14 days.. (d-f) The statistics results of second leaf sheath length (d), third leaf sheath length (e) or total length (f) of 14 days old seedlings grown under continuous dark, blue light (25 μmol $m^{-2}s^{-1}$), red light (25 μmol $m^{-2}s^{-1}$) or far-red light (15 μmol $m^{-2}s^{-1}$) conditions. Mean values ± s.d. ($n = 20$) are shown. Comparisons were performed by Student's *t*-tests (* $P < 0.05$, ** $P < 0.01$). (g-i) The statistics results of second leaf sheath length (g), third leaf sheath length (h) or total length (i) of 14 days old seedlings grown under continuous blue light with intensity of 0 to 35 μmol $m^{-2}s^{-1}$.

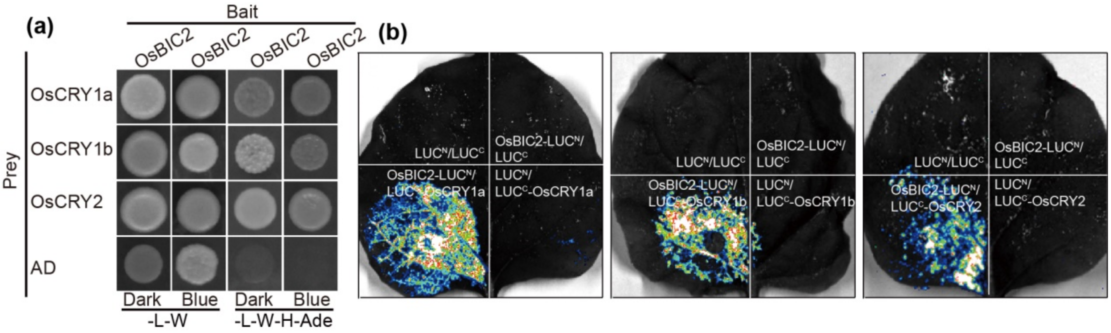

Figure S4. OsBIC2 interacted directly with OsCRYs. (a) OsBIC2 interacted with OsCRYs in yeast two-hybrid assays. Empty vector expressing the AD domain was negative control. (b) Interaction between OsBIC2 and OsCRYs under LD in LCI assays.

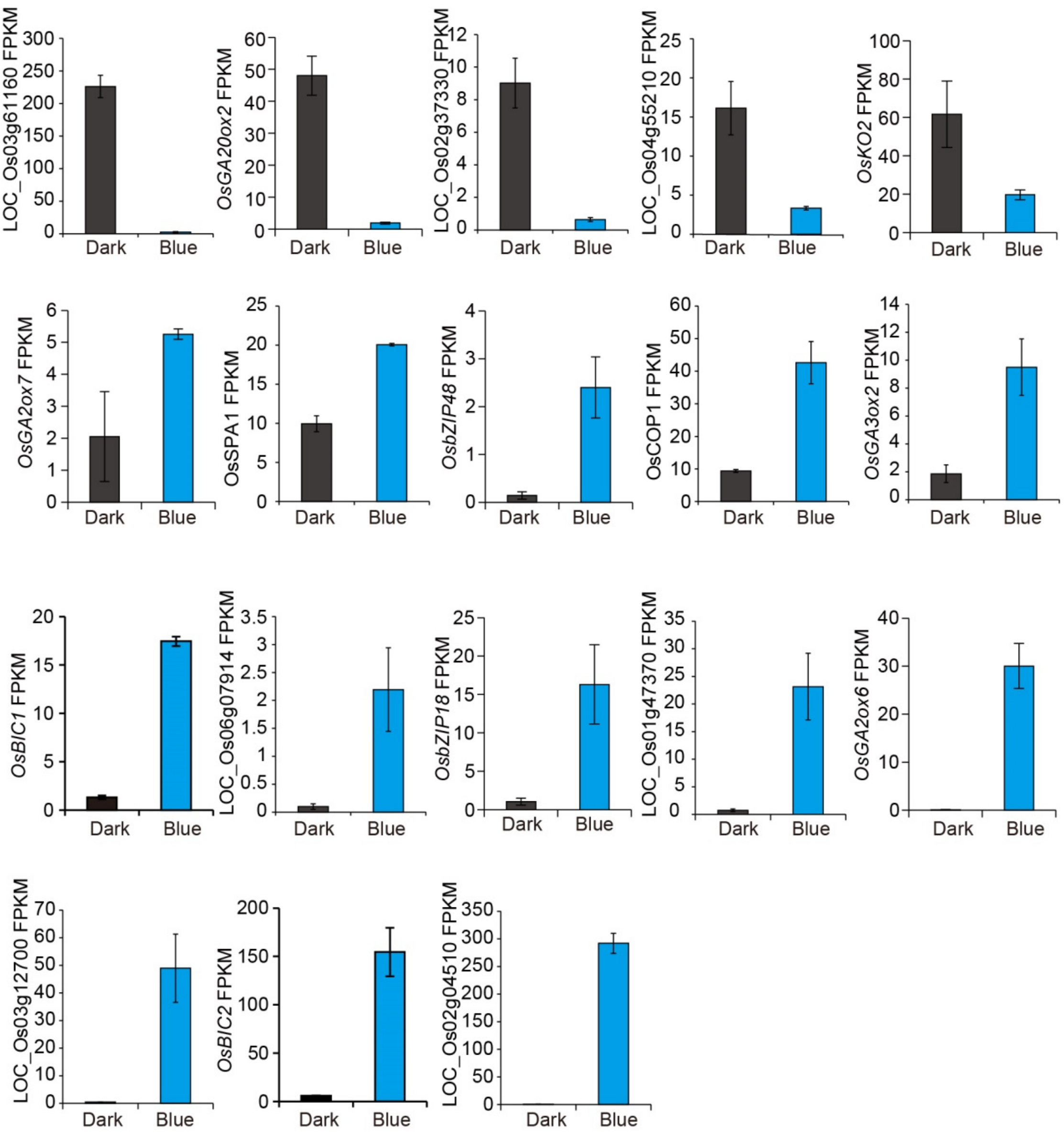

Figure S5. The FPKM analysis of 18 representative genes in WT under dark and blue light conditions by RNA-seq. Seven-day old etiolated seedlings were exposed to blue light (25 μmol $m^{-2}s^{-1}$) or kept in the dark for 2 hours.

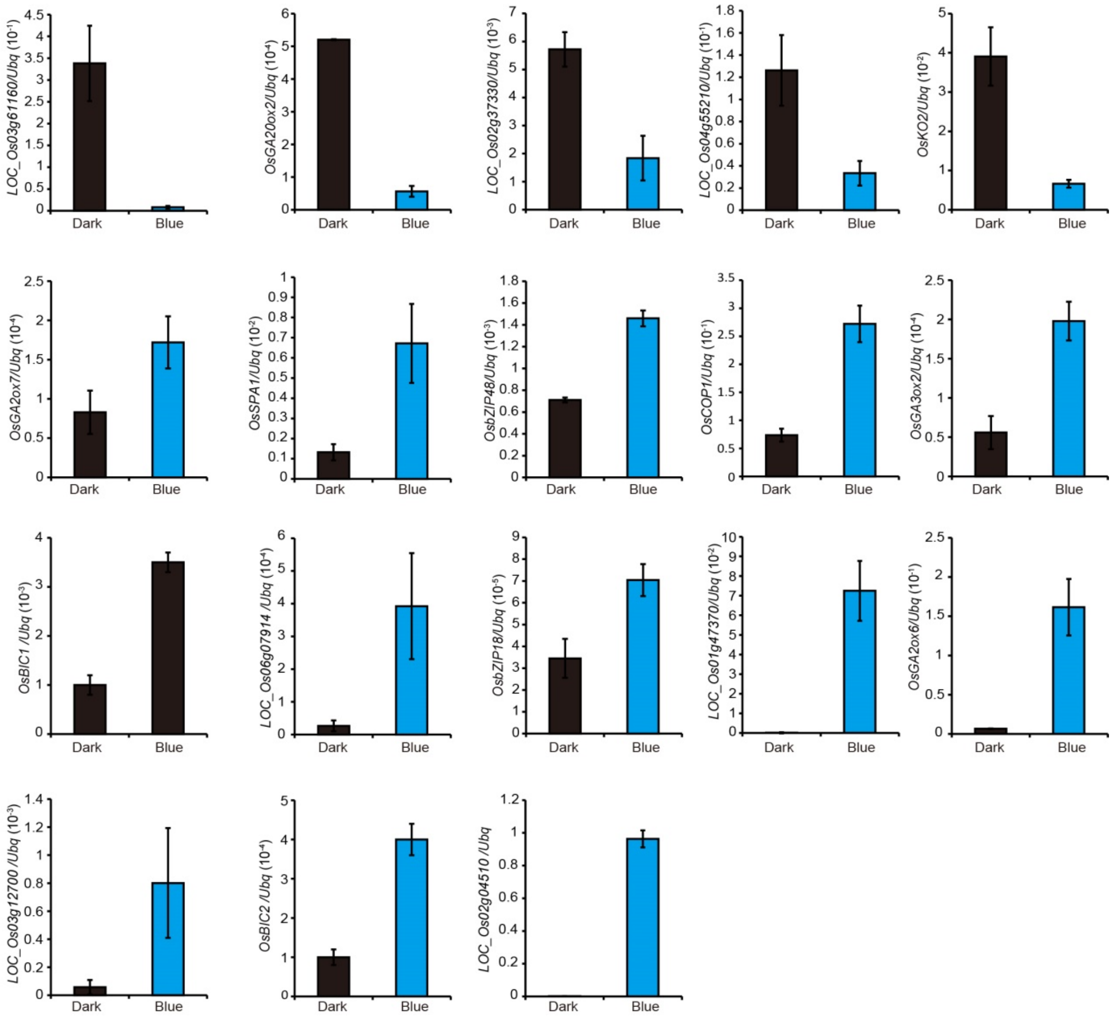

Figure S6. The transcriptional analysis of 18 representative genes in WT under dark and blue light conditions by qRT-PCR. Seven-day old etiolated seedlings were exposed to blue light (25 μmol $m^{-2}s^{-1}$) or kept in the dark for 2 hours.

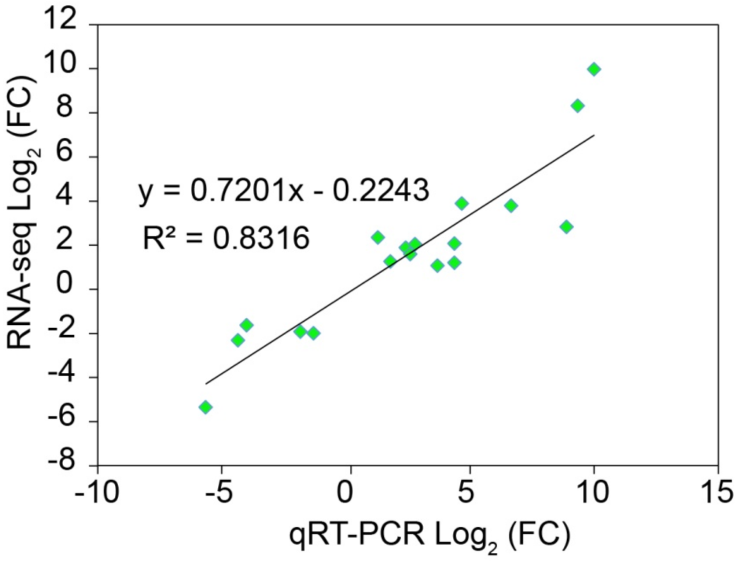

Figure S7. Correlation analysis of fold change (FC) of 18 representative genes in RNA-seq and qRT-PCR data. The x-axis field was the fold change (FC) of 18 representative genes in qRT-PCR data. The y-axis field was the fold change (FC) of 18 representative genes in RNA-seq data. The relationship between x-axis and y-axis was linear.
